# 纯化供者CD34^+^造血干细胞输注治疗异基因造血干细胞移植后移植物功能不良的回顾性临床分析

**DOI:** 10.3760/cma.j.cn121090-20241120-00460

**Published:** 2025-10

**Authors:** 硕 刘, 强 李, 振云 刘, 瑞辉 杜, 斌 刘, 兆勇 马, 尔烈 姜, 四洲 冯, 佳丽 孙

**Affiliations:** 1 中国医学科学院血液病医院（中国医学科学院血液学研究所），血液与健康全国重点实验室，国家血液系统疾病临床医学研究中心，细胞生态海河实验室，天津 300020 State Key Laboratory of Experimental Hematology, National Clinical Research Center for Blood Diseases, Haihe Laboratory of Cell Ecosystem, Institute of Hematology & Blood Diseases Hospital, Chinese Academy of Medical Sciences & Peking Union Medical College, Tianjin 300020, China; 2 天津医学健康研究院，天津 301600 Tianjin Institutes of Health Science, Tianjin 301600, China

**Keywords:** 造血干细胞移植, 移植物功能不良, 纯化供者CD34^+^细胞, 造血重建, Hematopoietic stem cell transplantation, Poor graft function, Donor purified CD34^+^ stem cells boost, Hematopoietic reconstruction

## Abstract

**目的:**

分析纯化供者外周血CD34阳性造血干细胞（以下简写为CD34^+^细胞）输注对异基因造血干细胞移植（allo-HSCT）后发生移植物功能不良（PGF）患者的疗效。

**方法:**

回顾性分析2019年9月至2023年3月期间在中国医学科学院血液病医院进行供者CD34^+^细胞分选并输注治疗的25例发生PGF患者的临床资料，其中半相合19例，全相合6例。利用免疫磁珠的方法分选纯化CD34^+^细胞用于患者的治疗性输注，以CD34^+^细胞的阳性率和回收率来分析纯化的效果，患者预后情况以造血恢复、总生存（OS）率以及移植物抗宿主病（GVHD）发生等指标进行分析。

**结果:**

分选纯化前后的CD34^+^细胞总数的中位值分别为2.64（0.82～6.53）×10^8^个和2.22（0.48～5.68）×10^8^个，中位回收率为78.37（58.48～115.72）％。纯化CD34^+^细胞输注后，10例中性粒细胞植入不良的患者中8例（80.0％）恢复（中性粒细胞绝对计数≥0.5×10^9^/L），中位恢复时间为21（10～40）d，21例血小板植入不良的患者中15例（71.4％）恢复（血小板计数≥20×10^9^/L），中位恢复时间为15（13～38）d。与输注前相比，仅3例（12.0％）患者在输注纯化CD34^+^细胞后发生GVHD，其中Ⅰ度急性GVHD 2例，局限性慢性GVHD 1例。中位随访14.47（0.23～41.63）个月，患者总体CD34^+^细胞输注后的OS率为（65.63±8.28）％，输注后17例患者存活，中位生存时间为19.07（0.23～41.63）个月。

**结论:**

利用免疫磁珠方法进行CD34^+^细胞纯化分选的效果良好，纯化的供者CD34^+^细胞输注可安全、有效地改善allo-HSCT后发生的PGF。

## Abstract (en)

**Objective:**

To evaluate the efficacy of purified donor CD34 positive hematopoietic stem cell（CD34^+^ cell）infusion in patients with poor graft function（PGF）after allogeneic hematopoietic stem cell transplantation（allo-HSCT）.

**Methods:**

The clinical data of 25 patients with PGF who underwent donor CD34^+^ cell sorting and infusion at the Institute of Hematology & Blood Diseases Hospital, Chinese Academy of Medical Sciences between September 2019 and March 2023, were retrospectively analyzed. The cohort included 19 haploidentical and 6 HLA-matched cases. CD34^+^ cells were purified using immunomagnetic beads for therapeutic infusion. The purification efficiency was evaluated based on the purity and recovery rate of CD34^+^ cells. Clinical outcomes were assessed by hematopoietic recovery, overall survival（OS）rate, and the incidence of graft-versus-host disease（GVHD）.

**Results:**

The median total number of CD34^+^ cells was 2.64（0.82–6.53）×10^8^ before purification and 2.22（0.48–5.68）×10^8^ after purification, with a median recovery rate of 78.37％（58.48％–115.72％）. After infusion of purified CD34^+^ cells, 8 of 10 patients（80.0％）with poor neutrophil engraftment achieved recovery（absolute neutrophil count ≥ 0.5×10^9^/L）, with a median time to recovery of 21（10–40）days. And 15 of 21 patients（71.4％）with poor platelet engraftment achieved recovery（platelet count ≥ 20×10^9^/L）, with a median time to recovery of 15（13–38）days. Only 3 patients（12.0％）developed GVHD after the infusion of purified CD34^+^ cells, including 2 cases of grade I acute GVHD and 1 case of limited chronic GVHD. With a median follow-up of 14.47（0.23–41.63）months, the overall OS rate after CD34^+^ cell infusion was（65.63± 8.28）％. Seventeen patients survived, with a median survival time of 19.07（0.23–41.63）months.

**Conclusion:**

Purification of CD34^+^ cells using immunomagnetic beads is effective, and the infusion of these purified donor CD34^+^ cells can safely and effectively improve PGF after allo-HSCT.

异基因造血干细胞移植（allo-HSCT）是恶性血液病患者的有效治疗方式，但部分患者的造血与免疫功能在移植后未恢复或恢复延迟，即移植物功能不良（PGF）。一般原发性PGF是指在移植后早期，即移植后28 d内，中性粒细胞绝对计数（ANC）低于0.5×10^9^/L，且这种情况与复发、短暂感染或药物不良反应无关[1]–[2]。继发性PGF是指在初次植入成功后，再次出现PGF，通常发生在移植28 d之后，并可能由多种因素引起，如感染、移植物抗宿主病（GVHD）等[3]。PGF的发生严重影响患者预后[4]–[5]，近年来利用免疫磁珠分选纯化CD34阳性造血干细胞（以下简写为CD34^+^细胞）后输注给患者治疗PGF的方法受到广泛关注，并取得了良好的临床治疗效果[6]–[10]。该方法纯化CD34^+^细胞的同时去除了移植物中绝大多数的CD3^+^ T细胞、CD19^+^ B细胞、自然杀伤（NK）细胞等淋巴细胞，很大程度地降低了输注后GVHD发生的概率[11]–[12]。

本研究统计我院进行亲缘供者CD34^+^细胞分选并输注治疗25例PGF患者的资料，比较分析了分选试验的效果及患者输注后造血恢复情况，以观察和衡量治疗效果。

## 病例与方法

1. 病例：本研究回顾性分析2019年9月到2023年3月期间在中国医学科学院血液病医院进行供者外周血CD34^+^细胞分选并输注的25例患者的临床资料。其中急性髓系白血病9例、骨髓增生异常综合征6例、再生障碍性贫血或（超）重型再生障碍性贫血6例、急性淋巴细胞白血病2例、慢性髓系白血病加速期1例、急性混合性白血病1例。患者进行首次allo-HSCT及本次供者CD34^+^细胞输注时移植物均来自同一供者。

所有患者在allo-HSCT后至CD34^+^细胞输注前细胞均为完全供者嵌合，且已排除因移植物排斥引起的移植失败（混合型嵌合体）、疾病复发等可能引起血细胞减少的其他因素，可确定诊断为PGF。本研究经中国医学科学院血液病医院伦理委员会批准（批件号：IIT2021011-EC-1），并已取得患者知情同意。

2. 供者的动员与采集：所有患者输注的纯化CD34^+^细胞均来自首次allo-HSCT时所对应的同一亲缘供者。应用G-CSF 250/300 µg每12小时皮下注射对供者进行外周造血干细胞动员，并在第5天或第6天采集外周造血干细胞。

3. CD34^+^细胞的分选纯化及检测：供者外周造血干细胞采集物中CD34^+^细胞的分选纯化采用CliniMACS Plus仪器（德国Miltenyi Biotec公司），首先将外周造血干细胞采集物与两倍体积的缓冲液（含磷酸盐和人血白蛋白）混匀并以200×*g*速度离心15 min去除血浆上清，接着加入CD34磁珠抗体，置于摇床上室温孵育30 min，然后再次以上述同样的方式加入缓冲液并离心洗去多余抗体，随后开启CliniMACS Plus仪器的分选程序，将与磁珠抗体结合的CD34^+^细胞吸附到分选柱上，最后洗脱收集；采用流式细胞术检测CD34^+^细胞和CD3^+^等免疫亚群细胞所占的比例，所用流式细胞仪型号为Canto Ⅱ（美国BD公司），抗体和溶血素试剂也均来自美国BD公司。CD34阳性率检测时应用DRAQ5荧光活性染料（美国Everbright公司）去除死亡细胞造成的影响，CD34^+^、CD3^+^等细胞成分的输注量根据流式细胞术所测得的比例及患者体重计算得出。分选回收率（％）=（分选前CD34^+^细胞总数/分选后CD34^+^细胞总数）×100％。

4. 造血重建与疗效评价：CD34^+^细胞输注后患者定期进行血常规检测，移植后连续3 d ANC≥0.5×10^9^/L的第1天为粒细胞植入时间，脱离血小板输注且连续7 d血小板计数（PLT）≥20×10^9^/L的第1天为血小板植入时间。

5. 随访：所有患者均通过门诊或住院病历以及电话联系随访至2023年9月30日，总生存（OS）期即从输注纯化CD34^+^细胞开始到随访结束或患者死亡的时间。

6. 统计学处理：应用SPSS 20.0对数据进行统计学分析，计数资料用例数（百分比）表示，细胞数、年龄及时间等计量资料以*M*（范围）表示，血细胞分析结果以均值±标准差表示，两组计量数据的比较采用独立样本*t*检验，生存率的分析采用Kaplan-Meier生存分析，*P*<0.05为差异有统计学意义。

## 结果

1. 患者一般特征及前期治疗情况：25例患者的一般特征见表1。所有患者首次allo-HSCT治疗时输注的均为亲缘供者的外周血采集物，移植时输注有核细胞（NC）总数的中位数为12.15（7.12～20.81）×10^8^/kg，CD34^+^细胞中位数为3.10（1.19～11.14）×10^6^/kg。患者均采用清髓性预处理方案，其中23例为白消安（Bu）+环磷酰胺（CTX）+氟达拉滨（Flu）/克拉屈滨（CLAD）+阿糖胞苷（Ara-C）+兔抗人胸腺细胞免疫球蛋白（rATG），1例为全身照射（TBI）+CTX+Flu/CLAD+Ara-C+rATG，1例为TBI+CTX+依托泊苷+rATG。GVHD的预防采用环孢素（CsA）或以他克莫司（FK506）为主，联合短疗程甲氨蝶呤的方案，半相合移植的患者同时联合霉酚酸酯。

**表1:** 异基因造血干细胞移植后发生移植物功能不良的25例患者的一般临床资料［例（％）］

特征	统计值
年龄［岁，*M*（范围）］	37（8～61）
性别	
男	16（64.0）
女	9（36.0）
供受者HLA配型	
HLA全相合	6（24.0）
HLA半相合	19（76.0）
供受者ABO血型	
相合	18（72.0）
不合	7（28.0）
供受者性别	
男供男	8（32.0）
男供女	6（24.0）
女供男	8（32.0）
女供女	3（12.0）

25例患者首次移植与纯化CD34^+^细胞输注的中位间隔时间为7.13（2.93～26.90）个月，在此期间，有19例患者出现不同程度的GVHD，8例患者发生巨细胞病毒血症，9例患者肺部感染，并且大部分患者（23例）在出现造血功能恢复不良的情况后进行过一定的药物或者细胞治疗，包括G-CSF或GM-CSF、TPO、艾曲泊帕或海曲泊帕、间充质干细胞（MSC）或脐血干细胞（CBSC）输注和冻存供者外周血干细胞（CPBSC）输注等方法的单独应用或者联合应用（表2），但症状未明显改善。

**表2:** 25例异基因造血干细胞移植后发生移植物功能不良的患者输注纯化CD34^+^细胞前的一般治疗方式

治疗方式	例（％）
无	2（8.0）
G-CSF/GM-CSF	1（4.0）
TPO±艾曲泊帕/海曲泊帕	11（44.0）
MSC/CBSC	1（4.0）
G-CSF/GM-CSF+TPO±艾曲泊帕/海曲泊帕	4（16.0）
G-CSF/GM-CSF+CPBSC	1（4.0）
G-CSF/GM-CSF+TPO±艾曲泊帕/海曲泊帕+MSC/CBSC/CPBSC	3（12.0）
TPO+艾曲泊帕/海曲泊帕+MSC/CBSC/CPBSC	2（8.0）

**注** G-CSF：粒细胞集落刺激因子；GM-CSF：粒细胞-巨噬细胞集落刺激因子；TPO：促血小板生成素；MSC：间充质干细胞；CBSC：脐血干细胞；CPBSC：冻存供者外周血干细胞

2. CD34^+^细胞分选效率：经检测，分选前后有核细胞（NC）总数分别为6.00（2.62～6.91）×10^10^个和2.18（0.62～6.13）×10^8^个。在加入活性染料DRAQ5去除死亡细胞的影响后，分选前后检测到的CD34^+^细胞比例分别为0.49％（0.21％～1.30％）和92.19％（75.60％～98.88％），计算得到CD34^+^细胞总数在分选前后分别为2.64（0.82～6.53）×10^8^个和2.22（0.48～5.68）×10^8^个，最终的CD34^+^细胞中位回收率为78.37％（58.48％～115.72％）。

此外，我们对23例患者的移植物样本在分选前后的各类免疫亚群细胞数量进行了对比（表3，其余2例患者因数据缺失未计入），发现总体淋巴细胞和包括T淋巴细胞、NK细胞在内的各淋巴细胞亚群、单核细胞以及DC1、DC2两类树突状细胞数量都降低了4个对数级。值得注意的是，与其他类型的细胞相比，CD19^+^ B淋巴细胞的数量在分选后只降低了3个对数级，提示分选后此类细胞残留更多。

**表3:** 23例异基因造血干细胞移植后发生移植物功能不良的患者各类免疫亚群细胞数量在分选前后的比较

细胞种类	分选前细胞总数 ［个，*Ｍ*（范围）］	分选后细胞总数 ［个，*Ｍ*（范围）］	分选后降低数量级
淋巴细胞	1.08（0.04～1.95）×10^10^	2.82（0.39～20.70）×10^6^	10^4^
CD3^+^ T淋巴细胞	7.77（0.08～14.20）×10^9^	2.87（0.17～12.86）×10^5^	10^4^
CD3^+^CD4^+^辅助性T细胞	3.71（0.02～5.77）×10^9^	1.27（0.002～3.27）×10^5^	10^4^
CD3^+^CD8^+^杀伤性T细胞	3.01（0.06～8.03）×10^9^	0.98（0.003～3.65）×10^5^	10^4^
CD16^+^CD56^+^ NK细胞	1.22（0.07～4.95）×10^9^	2.75（0.002～49.38）×10^5^	10^4^
CD19^+^ B淋巴细胞	1.60（0.08～2.96）×10^9^	1.84（0.18～19.77）×10^6^	10^3^
CD4^+^CD25^+^调节性T细胞	1.99（0.001～8.10）×10^8^	2.23（0.01～25.25）×10^4^	10^4^
lin^-^HLA-DR^+^ CD11c^+^树突状细胞亚群DC1	0.91（0.05～5.64）×10^8^	0.07（0.01～3.49）×10^4^	10^4^
lin^-^HLA-DR^+^ CD123^+^树突状细胞亚群DC2	1.12（0.05～4.80）×10^8^	0.12（0.01～4.52）×10^4^	10^4^
CD14^+^单核细胞	1.11（0.01～2.12）×10^10^	2.07（0.08～19.05）×10^6^	10^4^

**注** NK细胞：自然杀伤细胞；DC：树突状细胞

3. CD34^+^细胞输注后的造血恢复情况：患者输注前后基本情况如表4，输注纯化CD34^+^细胞前中性粒细胞植入不良10例（40.0％）、血小板植入不良21例（84.0％）、红系植入不良11例（44.0％）。纯化CD34^+^细胞输注后，中性粒细胞植入不良患者8例（80.0％）恢复（ANC≥0.5×10^9^/L），中位恢复时间为21（10～40）d，ANC由输注前的（0.46±0.33）×10^9^/L升高到（1.96±1.16）×10^9^/L，余2例未能植入成功。血小板植入不良患者输注后15例（71.4％）恢复（PLT≥20×10^9^/L），中位恢复时间为15（13～38）d，PLT由输注前的（11.25±6.44）×10^9^/L升高到（60.36±37.04）×10^9^/L，余6例未能植入成功。

**表4:** 纯化供者CD34^+^细胞输注的25例异基因造血干细胞移植后发生移植物功能不良患者情况及治疗预后

例号	性别	年龄（岁）	HLA配型	累及细胞系	首次移植与CD34^+^细胞输注间隔时间（月）	首次移植到CD34^+^细胞输注期间患者病情	CD34^+^细胞输注量（×10^6^/kg）	ANC恢复时间（d）	PLT恢复时间（d）	改善细胞系	随访时间（月）	生存情况
1	男	27	7/10	P	6.73	cGVHD（口腔、皮肤、眼），肺部感染，CMV血症	2.27	/	14	N+P+R	41.63	存活
2	男	49	8/10	P	5.27	cGVHD（皮肤、肠道），CMV血症，分选时CD34^+^细胞采集量低	0.73	/	18	N+P+R	9.07	死亡
3	男	13	8/10	N+P+R	10.80	cGVHD（皮肤），肝功能异常	2.79	26	14	N+P+R	37.60	存活
4	男	53	全相合	P	6.60	cGVHD（肝脏），分选时CD34^+^细胞采集量低	0.75	/	未植入	N+P+R	2.77	死亡
5	女	56	5/10	P+R	6.23	CMV血症，心功能不全，分选时CD34^+^细胞采集量低	0.55	/	未植入	N+R	9.93	死亡
6	男	52	6/10	P	15.60	cGVHD，肺部感染	1.69	/	未植入	无	0.37	死亡
7	女	28	5/10	P+R	7.50	aGVHD（Ⅰ度，皮肤2级，肠道1级），肝功能异常	6.69	/	17	N+P+R	30.33	存活
8	女	52	5/10	N+P+R	10.07	肺部感染，肝功能异常，硬膜下出血	4.85	20	13	N+P+R	20.63	病危
9	男	28	5/10	N+P+R	6.77	aGVHD（肺部、肝脏），CMV血症，肺部感染	6.06	17	未植入	N+P+R	28.03	存活
10	女	20	5/10	P+R	26.90	无	4.40	/	13	P+R	26.80	存活
11	男	20	全相合	P	4.70	CMV血症，肺部感染	5.09	/	14	N+P+R	25.23	存活
12	女	36	全相合	P+R	2.93	aGVHD（肝脏）	3.37	/	13	N+P+R	24.73	存活
13	男	48	5/10	P	9.00	aGVHD（Ⅲ度，肝脏3级，肠道4级），菌血症，脑梗死，电解质代谢紊乱	2.16	/	未植入	P	0.83	死亡
14	男	61	5/10	N	6.43	aGVHD（Ⅲ度，肠道），CMV血症，肠道感染，病毒脑炎	5.12	10	/	N	1.03	死亡
15	男	33	6/10	P	7.13	继发性骨髓纤维化，aGVHD（Ⅱ度，肠道1级）	2.52	/	15	无	19.07	存活
16	男	8	全相合	N+P+R	11.87	分选时CD34^+^细胞采集量低	0.97	25	30	N+P+R	18.63	存活
17	女	20	5/10	N	11.80	白血病髓外浸润，cGVHD（皮肤）	13.42	40	/	N+P+R	17.40	存活
18	女	14	5/10	N	18.23	cGVHD（皮肤）	2.78	15	/	R	17.03	存活
19	男	57	全相合	N+P	7.03	肺部感染，cGVHD（皮肤）	2.37	22	38	N+P+R	1.67	死亡
20	男	58	全相合	N+P+R	7.13	aGVHD（Ⅲ度，肠道4级，肝脏2级），心功能不全，谵妄，骨髓抑制	1.52	未植入	未植入	无	0.43	死亡
21	女	54	6/10	P	11.93	肝功能异常	3.22	/	20	P+R	14.47	存活
22	男	25	6/12	N+R	3.67	aGVHD（Ⅱ度，肠道1级），CMV血症	3.85	未植入	/	N+P+R	14.00	存活
23	男	55	6/12	P+R	5.03	aGVHD（Ⅲ度，皮肤2级，胃肠道4级），肺部感染	2.52	/	31	N+P+R	0.23	病危
24	女	23	8/12	P	6.60	aGVHD（Ⅲ度，肠道4级），肺真菌感染，肺出血，CMV血症	6.68	/	14	P	5.07	病危
25	男	44	5/10	P	20.10	aGVHD（Ⅳ度，皮肤，肝脏，肠道），难治性血小板减少，肺炎	3.28	/	17	N+P	5.37	存活

**注** ANC：中性粒细胞绝对计数；PLT：血小板计数；P：血小板；cGVHD：慢性移植物抗宿主病；CMV：巨细胞病毒；N：中性粒细胞；R：红细胞；aGVHD：急性移植物抗宿主病；/：该患者的中性粒细胞或血小板未被累及，故未统计恢复时间

根据25例PGF患者所累及细胞系的不同，将患者分为血小板/血小板+红细胞（P/P+R）组（15例）、中性粒细胞/中性粒细胞+红细胞（N/N+R）组（4例）、中性粒细胞+血小板/中性粒细胞+血小板+红细胞（N+P/N+P+R）组（6例）进行了比较。血小板恢复的患者比例较中性粒细胞恢复少，其中死亡或病危的患者P/P+R组为7例（7/15，46.7％），N+P/N+P+R组为3例（3/6，50.0％），N/N+R组最少，仅有1例（1/4，25.0％）。P/P+R组的中位随访时间最短，为9.93（0.23～41.63）个月；N/N+R和N+P/N+P+R两组的中位随访时间分别为15.52（1.03～17.40）个月和19.63（0.43～37.60）个月。从细胞系的恢复时间来看，同时累及中性粒细胞和血小板的N+P/N+P+R组恢复的中位时间相对较长，中性粒细胞恢复中位时间为22（17～26）d，血小板恢复中位时间为22（13～38）d。

为观察纯化CD34^+^细胞输注后对患者血象恢复所起的作用，对输注前后25例患者的静脉血血常规数据进行比较。输注前后25例患者的ANC分别为（1.22±0.84）×10^9^/L和（2.10±1.38）×10^9^/L，其中17例有不同程度的升高，3例降低，5例无明显变化。输注前后PLT为（16.16±12.01）×10^9^/L和（47.32±36.03）×10^9^/L，其中19例有不同程度的升高，2例降低，4例无明显变化。输注前后血红蛋白分别为（76.72±17.50）g/L和（89.88±23.28）g/L，其中18例有不同程度的升高，2例降低，5例无明显变化。总体而言，纯化CD34^+^细胞输注后患者的ANC、PLT及HGB都有明显改善且差异均具有统计学意义（*P*值分别为0.037、0.002、0.029）。

4. 患者预后及OS情况：输注纯化CD34^+^细胞后，患者GVHD的发生情况与输注前相比基本无变化，仅有3例（12.0％）患者发生不同程度的GVHD，其中2例为Ⅰ度急性GVHD（分别是皮肤Ⅱ级和肝脏Ⅰ级），1例发生局限性慢性GVHD（皮肤、口腔、眼）。

至随访截止日期，患者总体中位随访时间为14.47（0.23～41.63）个月，纯化CD34^+^细胞输注后的25例患者OS率为（65.63±8.28）％，其中3例在院时间不足1个月。输注后共有17例患者存活，中位生存时间为19.07（0.23～41.63）个月。有8例患者在输注后死亡，这8例患者的中位生存时间为1.35（0.37～9.93）个月，其中2例患者死于多脏器功能衰竭、3例死于心功能不全、1例死于重症感染，1例死于肝性脑病导致的心衰昏迷，1例患者在末次复查时状况良好，但之后电话随访时家属告知患者已死亡。

## 讨论

PGF是allo-HSCT治疗过程中较为常见的并发症，发生率为5％～27％。PGF的发生与移植前患者疾病状态、HLA配型、GVHD、药物不良反应及病毒感染等多种因素有关[1],[4],[13]，一旦发生，将导致较高的发病率和死亡率，严重威胁患者的生命健康。

通过减停骨髓毒性药物或者利用生长因子（如G-CSF）刺激造血等传统方法治疗PGF的效果十分有限[3]–[4],[14]，而进行完全二次移植则可能因移植物未经处理而导致非复发死亡率（NRM）升高或严重GVHD的发生[15]–[17]。因此，应用免疫磁珠分选纯化CD34^+^细胞后再输注的治疗方法得到广泛应用[7]–[10]。由于操作过程中可去除大部分可引起免疫应答的免疫细胞（如T细胞、B细胞、NK细胞等），该方法在很大程度上降低了患者GVHD发生率[18]–[19]。

本研究对免疫磁珠分选前后移植物中的CD34^+^细胞及各免疫亚群细胞数量的统计显示，分选后的阳性率（92.19％）和回收率（78.37％）较为理想[11]–[12],[20]。由于个别供者的采集物中NC数偏高，在分选前检测CD34^+^细胞比例时受此影响所得结果偏低，因此最终计算所得的回收率超过了100％。其他类型细胞的数量都在分选后降低了10^4^个数量级，而CD19^+^ B细胞的数量在分选后只降低了10^3^个数量级，相对残留较多。这可能是因为CD34^+^CD19^+^B淋巴细胞前体细胞的存在[21]–[22]，需要进一步研究。

本研究结果显示，纯化CD34^+^细胞输注可有效促进PGF患者中性粒细胞和血小板的造血恢复，有些患者输注后虽然未达到植入标准，但三系细胞都有不同程度的升高。少数患者造血功能仍未恢复，可能与其CD34^+^细胞的输注量较低（<2×10^6^/kg）有关，但也有报道显示两者没有必然的相关性。费新红等[23]的回顾性研究显示部分患者回输的纯化CD34^+^细胞数量仅为1.1×10^6^/kg且CD34^+^细胞纯度为44％，但取得良好的治疗效果。Cuadrado等[24]的多因素分析结果显示患者血细胞计数恢复的相关因素包括患者及供者血清CMV阴性、无活动性感染以及患者和供者的性别匹配。另外，根据患者累及细胞系不同的分组比较可以看出，累及血小板的血小板未恢复患者例数以及死亡或病危例数较多。但由于本研究例数较少，需更多的数据积累来证明。

在纯化CD34^+^细胞输注治疗之前，本研究中23例（92％）患者曾接受过其他方式的治疗，包括G-CSF、TPO、艾曲泊帕或海曲泊帕、MSC或CBSC输注等方法的单独或联合应用，但这些治疗方式的应用存在局限性。例如，MSC输注虽然在改善免疫耐受和GVHD的预防与治疗方面具有一定的优势，但在促进造血恢复方面可能不如纯化CD34^+^细胞直接有效[25]–[26]。CBSC由于较难获取，且获得的细胞数低[27]，通常在造血干细胞移植中只是作为辅助性输注。这些方法虽未表现出明显的积极作用，但与CD34^+^细胞输注是否存在协同效应尚未可知。大量研究表明[28]–[30]，G-CSF和TPO可以通过影响造血干细胞的存活、增殖和分化来促进造血恢复，而MSC则具有免疫调节、造血支持和促进组织修复的作用，这些都可能有助于输注到患者体内的CD34^+^细胞更好地发挥作用，值得进一步研究探讨，以便为患者寻找更佳的治疗方案。

本研究中，患者输注纯化CD34^+^细胞后GVHD的发生率较低，仅有3例患者发生轻度的GVHD。比较发现，这3例患者的淋巴细胞输注量相对较高，这印证了去除移植物中的免疫细胞可明显降低患者GVHD的发生率。Askaa等[31]的研究也显示，较低的GVHD发生率与分选后移植物中的CD3^+^ T细胞数量少有关。

另外，利用纯化供者CD34^+^细胞治疗PGF的应用领域也有待拓展。如纯化CD34^+^细胞可与CAR-T细胞治疗相结合[32]–[33]，移植物中CD3^+^ T细胞会增加GVHD的发生风险，但这类细胞也可避免患者发生感染性并发症并发挥移植物抗白血病的作用[34]–[35]，因此需要更大的样本量研究来确定合适的细胞输注量。另外，Ghobadi等[36]的研究表明在无法获得新鲜采集的干细胞的情况下，冷冻保存的干细胞可作为替代物，分选纯化出CD34^+^细胞后输注给PGF患者进行治疗。

本研究证实了纯化供者CD34^+^细胞输注能够在低GVHD发生率的前提下有效帮助PGF患者造血重建，而对于治疗过程中的各种影响因素与患者最终恢复效果之间的关联，比如细胞输注前的用药方案、CD34^+^细胞输注量等，还需要更多的研究来进行验证。
